# Stress resistance and lifespan enhanced by downregulation of antimicrobial peptide genes in the Imd pathway

**DOI:** 10.18632/aging.101417

**Published:** 2018-04-19

**Authors:** Yuh-Ru Lin, Hardik Parikh, Yongkyu Park

**Affiliations:** 1 Department of Cell Biology and Molecular Medicine, Rutgers-New Jersey Medical School, Newark, NJ 07103, USA; 2 Present address: Institute of Molecular and Genomic Medicine, National Health Research Institutes, Zhunan, Miaoli 35053 Taiwan; 3 Present address: Institute of Ophthalmology and Visual Science, Rutgers, State University of New Jersey, Newark, NJ 07103, USA

**Keywords:** flies developed at 18°C, Imd pathway, AMP genes, stress resistance, lifespan

## Abstract

Biological behaviors and longevity of ectothermic animals are remarkably influenced by ambient temperature. Development at 18°C significantly enhances the stress resistance of adult flies with more accumulation of nutrients (especially fat) in the body than development at 25°C. Gene expression analysis between the flies developed at 18°C and 25°C revealed that the Immune deficiency (Imd) pathway, including the downstream antimicrobial peptides (AMPs), is downregulated in the flies developed at 18°C. When hypomorphic *imd* mutant flies with reduced AMP expressions were developed at 25°C, they showed induced stress resistance with higher fat content in the body similar to the wild-type flies developed at 18°C. However, severe hypomorphic *imd* mutants could not enhance stress resistance due to the downregulation of another downstream JNK pathway that expresses stress tolerance genes. Interestingly, the downregulation of AMP genes, itself, extended lifespan with increased stress resistance. Especially, fat body-specific downregulation of Imd AMP genes exhibited a longer lifespan with higher heat resistance. The fat body is known to function in metabolic homeostasis, stress tolerance, growth, and longevity in *Drosophila*. Here, we provide the first evidence that mild downregulation of the Imd pathway with AMP genes increases fat content, stress resistance, and lifespan in adult flies.

## Introduction

Fruit flies, which are ectothermic animals, can live more than twice as long at 18°C than at 25°C [1]. Even though it has been thought that this enhanced longevity at a lower temperature (18°C) is caused by a change of metabolic rate [2], the mechanisms that regulate longevity by ambient temperature are poorly understood. Previously, we found that development at 18°C (from embryo to newborn adult) significantly enhances stress resistance of adult flies with more accumulation of nutrients (especially fat) in the body than development at 25°C [3]. This enhanced resistance to stress was similarly observed in both sexes and sustained up to 30 days (middle age) after hatching of the adult flies [3], indicating that development at a lower temperature, 18°C, significantly enhances the mechanism(s) of stress resistance. Higher stress resistance and/or fat accumulation are frequently found in long-lived flies [4-6] such as mutants of the IGF (insulin/insulin-like growth factor) signaling pathway [7,8]. From the RT-PCR tests of representative stress-related genes, we showed that the development at a lower temperature (18°C) downregulates antimicrobial peptide genes, *AttA* and *DptB*, of the Immune deficiency (Imd) pathway [3]. The Imd pathway is known to regulate innate immune responses in *Drosophila* [9], and the Imd protein activates two downstream branches, JNK/basket and NF-kB/Relish, which are subsequently responsible for the upregulation of stress tolerance and antimicrobial peptide genes, respectively [10,11].

The roles of the Imd pathway have been well studied in a humoral response against intruders, which is characterized by the secretion of antimicrobial peptides (AMPs) into the hemolymph [12]. However, whether the Imd pathway is involved in a longevity mechanism has not been reported. Using hypomorphic *imd* and *AttC* mutant flies, here, we show that the mild downregulation of the Imd pathway has a beneficial effect for stress resistance with higher fat content in the body even when developed at 25°C. The Imd pathway functions for the immune response in the fat body [13] which is involved in the metabolism and storage of fat in adult flies. Surprisingly, our data show that the fat-body-specific downregulation of Imd AMP genes significantly enhances heat resistance and extends lifespan.

## RESULTS

### Imd downregulation in flies developed at 25°C enhances stress resistance and extends lifespan with reduced AMP expressions

**Figure 1:**
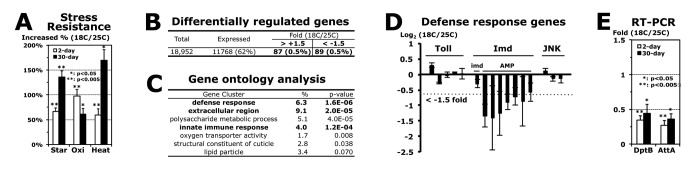
**Imd AMP genes are downregulated in adult flies developed at 18°C.** (**A**) Increased stress resistance of 2-day-old (white) and 30-day-old (black) male adult flies developed at 18 °C, which were compared from 2- and 30-day-old flies developed at 25 °C (0%), respectively. The median survival times of flies under each stress (starvation, oxidation or heat) were calculated from the survival curves of 3 ~ 5 independent experiments, and then the changed percentage is represented as average ± standard error of mean (SEM) following normalization with the median of flies developed at 25 °C (starvation: 28.2 and 11.1 hours; oxidation: 15.4 and 8.0 hours; heat: 15.3 and 1.7 hours of 2- and 30-day-old flies, respectively). P-value (*): Student’s t-test. (**B**) The gene expression analyses between 2-day-old male flies developed at 18°C and 25°C. From three independent microarray experiments, the fold changes of gene expression (18C/25C) were averaged with SEM. (**C**) With the genes changed more than 1.5 fold (total 176 in **B**), the gene ontology was analyzed using a DAVID web tool (http://david.abcc.ncifcrf.gov/home.jsp). %: involved genes/total 176 genes; p-value: a modified Fisher Exact. (**D**) Expressional changes of genes in Toll (*Tl*, *Def*, *Drs-l*, *Drs*, *Mtk*), Imd (*imd*, *DptB*, *AttA*, *AttB*, *AttC*, *CecB*, *CecC*, *Dro*) and JNK (*bsk*, *GstD1*, *Thor*) pathways from the microarray experiments (**B**). (**E**) RT and real-time PCR analyses of AMP genes in Imd pathway. Gene expression folds (18C/25C) of 2- and 30-day-old male flies were averaged from 4 ~ 8 independent experiments using four different RNA batches.

To investigate how stress resistance in flies is influenced by the developmental temperatures, we compared the RNA expression levels between 2-day-old male flies developed at 18°C and 25°C by microarray analyses (Fig. 1). We first filtered out non-expressed genes that received both of Affymetrix Absent calls in the gene expression of 18°C and 25°C development and, next, removed highly variable genes for which the standard error was bigger than 1.32-fold from three independent experiments. The final 62% of the genes (11,768) among total gene probes (18,952) were considered to have statistically reliable expressions in the 2-day-old male flies developed at 18°C and 25°C (Fig. 1B). With a cut-off value of ± 1.5-fold changes [14-16], we found that 0.9% of the genes (176/18,952) are differentially expressed between the flies developed at 18°C and 25°C (Fig. 1B). The gene ontology analyses of these 176 genes revealed a possible link of those genes to the stress resistance and aging process, such as defense responses, extracellular proteins, and the polysaccharide metabolic process (Fig. 1C). Among the defense response genes, antimicrobial peptide (AMP) genes in the Imd pathway [9] were specifically downregulated with the development at a lower temperature 18°C (Fig. 1D). Imd AMP genes, *DptB* and *AttA*, were still downregulated even after 30 days of age at 25°C (Fig. 1E) when the higher stress resistance was sustained (Fig. 1A).

**Figure 2:**
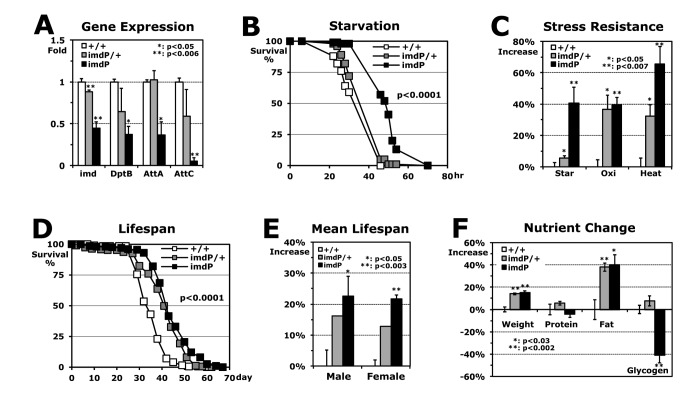
**Stress resistance and lifespan enhanced by Imd downregulation in flies developed at 25°C.** (**A**) RT-PCR analyses of *imd* and AMP genes in Imd pathway. Using total RNAs of 2-day-old male flies developed at 25°C, gene expressions in hetero (*imdP*/+) and homozygous (*imdP*) mutants were normalized with those of wild-type (+/+: 1 fold) and then, were averaged with SEM. (**B**) The survival curve for starvation stress using 2-day-old male flies (p-value: log-rank test). (**C**) Increased stress resistance of 2-day-old *imdP* mutant flies from the wild-type flies (+/+: 0%). The median survival times of flies under each stress (starvation, oxidation or heat) were calculated from the several survival curves (**B**) and then, the changed percentages are represented as average ± SEM after normalization with the median times of wild-type flies (Table 1). (**D**) The lifespan of adult male flies between the wild-type and *imdP* mutants. (**E**) Increased mean lifespan of *imdP* mutant flies. Percent changes of mean lifespan are indicated as average ± SEM normalized by wild-type lifespan days (Table 1), which were calculated from the several lifespan curves (**D**). (**F**) Changes of weight and nutritional contents between 2-day-old wild-type and *imdP* mutants male flies. After fresh weight per fly was measured (mg/fly), the nutrient contents were normalized by fresh weight of fly (μg/mg). The data represent average ± SEM normalized by nutrient contents of wild-type flies (Table 1).

To test if the downregulation of *imd* (0.76-fold) and AMP genes contributed to higher stress resistance in the flies developed at 18°C (Fig. 1A), we reduced the expression of the *imd* gene with the hypomorphic *imd* mutants and compared the stress resistance between the wild-type and *imd* mutant flies, both developed at 25°C. The *imdP* mutant, in which a P-element is inserted into the *imd* promoter, reduced the *imd* expression (heterozygote *imdP*/+: 0.88-fold; homozygote *imdP*: 0.45-fold in Fig. 2A) and then decreased the expression of downstream AMP genes (Fig. 2A). Dependent on the reduced levels of *imd* and AMP expressions, the stress resistances were enhanced (Fig. 2B-C) with a higher SOD2 (MnSOD) activity (33.8% increase). Interestingly, lifespan of *imdP* male flies were extended up to 24.8% (Fig. 2D) and these longer lifespans were shown in both male and female flies (Fig. 2E). Previous studies have suggested that attaining higher resistance against stresses may extend the lifespan of fruit flies [17-19]. It was shown that several long-lived mutant flies are more resistant to stresses such as starvation, oxidation, and heat than wild-type flies [5], indicating that lifespan and stress resistance often correlate positively with each other [20]. When we measured the nutrients of fly bodies, only fat (triacylglycerol) content increased ~40% in both hetero and homozygous *imdP* flies (Fig. 2F), which is commonly observed in long-lived mutant animals including *C. elegans*, flies, and mice [7,8,21]. These nutrient storages are often interpreted as an adaptation mechanism for increased energy demands during a longer lifespan and against stress resistance [6,22].

### Mild downregulation of the Imd pathway increases stress resistance and fat content in adult flies developed at 25°C

**Figure 3:**
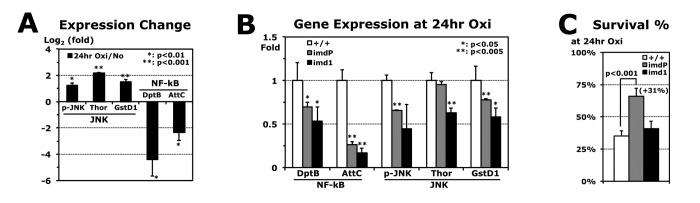
**Reduced AMP expressions during paraquat-induced oxidative stress.** (**A**) Gene expressions in 2-day-old male wild-type (+/+) flies after 24 hours of oxidative stress (20 mM paraquat), which are normalized from those of flies without the stress (log_2_1 = 0). p-JNK: average with SEM from Western blots (Phospho-JNK/GAPDH); Thor, GstD1 (JNK pathway), DptB, and AttC (NF-kB pathway): RT-PCR experiments using total RNAs of flies developed at 25°C. (**B**) Gene expressions in *imdP* and *imd1* homozygous mutants after 24 hours of oxidative stress, which were compared with those of wild-type (+/+: 1 fold). (**C**) Survival percentages of wild-type (+/+), *imdP*, and *imd1* mutants at 24 hours of oxidative stress. Parenthesis: increased survivorship (%) of *imdP* mutants from wild-type flies.

The *imd1* mutant is known to be a stronger hypomorphic mutant than *imdP* in the immune response to *E. coli* infection [23]. Although the expression change of the *imd* gene was not shown due to the missense mutation of *imd1* [24], the AMP genes in the Imd pathway were downregulated in the *imd1* flies (Table 1). The heterozygous (*imd1*/+) flies exhibited enhanced stress resistances, higher MnSOD activity, longer lifespans, and increased fat content (Table 1), as shown in the *imdP* mutants (Fig. 2). However, in *imd1* homozygous mutants, the stress resistance was not significantly changed under oxidation or was highly decreased when subjected to heat stress (Table 1). Another *imdNP* mutant, in which a P-element is inserted into the coding region of the *imd* gene, also showed the different phenotypes between the heterozygous and homozygous *imdNP* flies (Fig. S1). A possible explanation is that the JNK/basket pathway, which is activated by the Imd protein and then is subsequently responsible for upregulation of the stress response genes [10,11], is less active due to the severe loss of *imd* function. The JNK downstream genes, Thor and GstD1, are upregulated for stress tolerance [25]. As expected, their expressions were increased during oxidative stress (24 hours) of the wild-type flies (Fig. 3A). In contrast, AMP genes (DptB and AttC) were downregulated (Fig. 3A), which suggests that reducing Imd AMP expressions may be beneficial for stress tolerance. Compared to the wild-type flies, both *imdP* and *imd1* mutant flies show more reduced DptB and AttC expressions after 24 hours of oxidative stress (Fig. 3B). However, the *imd1* flies also showed significantly reduced expressions of Thor and GstD1 (Fig. 3B). Then, we found that the *imdP* flies were able to survive longer than the wild-type and *imd1* flies under the oxidative stress (Fig. 3C), probably due to reduced AMP expressions, but still sustained expressions of Thor and GstD1.

**Table 1:** The comparison of *imd1* hetero and homozygous mutants from the wild-type (+/+).

Genotype	Gene Expression (fold)					Stress Resistance (hr)					MnSOD	Lifespan (day)		Weight	Nutrients (μg/mg)			
	imd	DptB	AttA				Starvation	Oxidation	Heat	(mU/μg)		Male	Female	(mg/fly)		Protein	Fat	Glycogen	
+/+	1.00	1.00	1.00				31.8	22.8	13.7	0.30		35.1	39.3	0.67		56.2	11.1	18.7	
imd1/+	1.13	*0.55*	*0.48*				*46%*	**29%**	6%	*54%*		*17%*	*16%*	6%		-4%	*40%*	*32%*	
imd1	0.88	*0.34*	*0.32*				**37%**	7%	*-177%**	ND		*-34%**	ND	8%		-2%	**58%**	*-55%**	

The data of gene expression (RT-PCR), stress resistance (median survival time), MnSOD (activity per mitochondrial proteins), lifespan (mean lifespan), weight (fresh weight per fly), and nutritional contents (amount per fresh fly weight) represent the original values of wild-type and percentages changed from the wild-type in the *imd1* mutants (2-day-old male flies developed at 25°C). Bold: significant p-values < 0.05 (*) ~ 0.005 (**).

### AMP downregulations of the Imd pathway directly enhance stress resistance and extend lifespan

**Figure 4:**
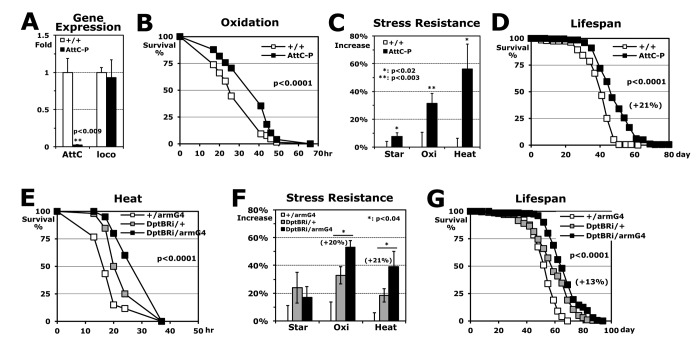
**Stress resistance and lifespan enhanced by downregulation of AMP genes in Imd pathway.** (**A**) Gene expressions of *AttC* and *loco* in *AttC-P* homozygous mutant, which were normalized with those of wild-type (+/+: 1 fold) using total RNAs of 2-day-old male flies developed at 25°C. (**B**) The survival curve for oxidation stress using 2-day-old male flies (p-value: log-rank test). (**C**) Increased stress resistance of 2-day-old *AttC-P* mutant flies from the wild-type flies (+/+: 0%). The median survival times of flies under each stress (starvation, oxidation or heat) were calculated from the several survival curves (**B**) and then, the percentages changed from wild-type flies are represented as average ± SEM. (**D**) The lifespan of adult male flies between the wild-type and *AttC-P* mutant. Parenthesis: increased percentage of mean lifespan of *AttC-P* mutant from the wild-type flies. (**E**) The survival curve for heat stress using 2-day-old male flies between single transgene controls (+/armG4, DptBRi/+) and DptB downregulation in a whole body (DptBRi/armG4). (**F**) Increased stress resistance of 2-day-old DptBRi/armG4 flies from the control +/armG4 (0%). Parenthesis: increased percentage of median survival time of the DptBRi/armG4 flies from another control DptBRi/+. (**G**) The lifespan of adult male flies between the two controls and DptB downregulation. Parenthesis: increase percentage of DptBRi/armG4 flies from the DptBRi/+ control.

Next, to investigate if the expression changes of Imd-driven AMP genes are directly related to stress resistance and longevity, we tested *AttC-P* mutant flies in which *AttC* expression is completely removed without any change in the expression of another longevity-related gene, *loco* [26] (Fig. 4A). The *AttC-P* flies interestingly enhanced stress resistances against oxidation and heat, significantly (Fig. 4B-C), as did the *imdP* mutant flies (Fig. 2C). In addition, lifespan was 21% extended with the *AttC* downregulation (Fig. 4D). Using an UAS/Gal4 system [27], we examined the downregulation effect of another AMP gene (UAS-*DptB*-dsRNAi: *DptB*Ri) with an *arm*-Gal4 driver that expresses a target UAS-gene in the whole body. The *DptB*Ri/*arm*Gal4 flies also exhibited higher stress resistance (~21% increase) and longer lifespan (+13%) compared to the control *DptB*Ri/+ flies (Fig. 4E-G), indicating that the expression levels of antimicrobial peptide genes in the Imd pathway are directly involved in the stress resistance and the longevity mechanism.

**Figure 5:**
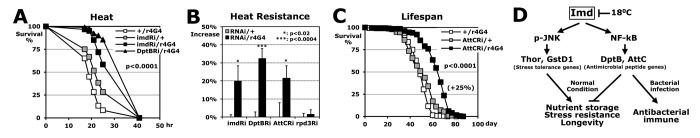
**Stress resistance and lifespan enhanced by fat body-specific downregulations of *imd* and AMP genes in Imd pathway.** (**A**) The survival curve for heat stress using 2-day-old male flies between single transgene controls (+/r4G4, imdRi/+) and fat-body-specific *imd* or *DptB* downregulation (imdRi/ or DptBRi/r4G4). (**B**) Comparison of heat resistance between RNAi single transgenic (Ri/+: 0%) and fat body-specific downregulation (Ri/r4G4). The median survival times of flies under heat stress were calculated from the several survival curves (**A**) and the median survival time of common control +/r4G4 flies was lower than those of each Ri/+ flies (**A**). (**C**) The lifespan of adult male flies between the two controls and a fat-body-specific *AttC* downregulation (AttCRi/r4G4). Parenthesis: increase percentage of the AttCRi/r4G4 from AttCRi/+ flies. (**D**) Imd pathway regulates the longevity mechanism through two pathways.

The fat body, the fruit fly analogue of mammalian liver and adipose tissues, is known to function in metabolic homeostasis, stress tolerance, growth, and longevity in *Drosophila* [28,29]. For the antimicrobial response to bacterial infection, the antimicrobial peptide genes are mainly synthesized from fat body and secreted into the hemolymph [13]. We tested how downregulation of AMP genes in the fat body affects stress resistance and lifespan. We found that fat-body-specific (r4-Gal4 [30],) downregulations of *imd*, *DptB,* and *AttC* genes enhanced heat resistances significantly, between 20% and 32% (Fig. 5A-B), compared to the downregulation of another longevity gene, *rpd3* [31,32], in the fat body (1.5% in Fig. 5B). Surprisingly, the *AttC*Ri/r4Gal4 flies could exhibit up to 25% extended lifespan compared to the control *AttC*Ri/+ flies (Fig. 5C), indicating that a manipulation of AMP expression in fat body tissues affects longevity of the whole body. Our data propose that the Imd pathway regulates the longevity mechanism through two downstream branches (Fig. 5D): 1) positively by stress tolerance genes of p-JNK [25,29,33,34] and 2) negatively by antimicrobial peptide genes of NF-kB that function positively in the immune response to bacterial infection [9,35].

## DISCUSSION

The antimicrobial peptide (AMP) genes in the Imd pathway were specifically downregulated in the wild-type flies developed at a lower temperature 18°C (Fig. 1D), which exhibited the stronger resistance to all three applied stressors (starvation, oxidation, and heat) (Fig. 1A) [3]. We found that the flies developed at 25°C also can enhance stress resistances (Fig. 2B-C) when the *imd* and downstream AMP genes are downregulated (Fig. 2A). However, these additional stress resistances by the *imdP* mutation in development at 25°C were not observed when the *imdP* flies were developed at 18°C (Fig. S2), indicating that the stress resistances are already induced by the downregulation of Imd AMP genes in wild-type flies developed at 18°C (Fig. 1). Instead, a heat resistance was significantly reduced in the *imdP* flies developed at 18°C (Fig. S2B) as shown in the severe hypomorphic *imd1* flies (Table 1). It implies that intense downregulation of the Imd pathway has a negative effect on the heat resistance with additional downregulation of another downstream JNK/basket pathway (Fig. 3B) that is required to activate the stress response genes [10,11]. Overall, our data support that a mild downregulation of the Imd pathway enhances stress resistance. However, the increased levels of stress resistance by *imdP* flies developed at 25°C (Fig. 2B-C) were not enough to reach the levels that the 18°C development of wild-type flies induces (Fig. 1A), suggesting that the downregulation of Imd AMP genes alone cannot fully explain the higher stress resistances in flies developed at 18°C (Fig. 1A).

Long-lived mutant animals, including *C. elegans*, flies, and mice, commonly show increased fat content [7,8,21], and our *imd*-downregulated (*imdP* and *imd1*/+) flies also show increased fat (triacylgycerol) content with extended lifespan (Fig. 2D-F and Table 1). It is known that Imd-driven AMP proteins dominantly function in the fat body, which is involved in the metabolism and storage of fat in adult flies, for the antimicrobial response to bacterial infection [13]. The increased fat content in *imd* flies suggests a possibility that Imd AMP proteins are related to the fat metabolism in *Drosophila* fat body. Accordingly, the fat-body-specific *AttC* downregulation (*AttC*Ri/r4Gal4) extended lifespan with enhanced heat resistance (Fig. 5). However, the Imd AMP downregulation in fat body did not affect starvation and oxidation resistances (data not shown) in contrast to the increased oxidation resistance shown with the downregulation in the whole body (Fig. 4B-C and F), suggesting that Imd AMP proteins may be involved differently in the several mechanisms against the stressors.

The *Drosophila* innate immunity AMP genes are upregulated during aging [15], which may prepare pathogen defenses promptly in the old-aged flies that have weak immune systems. However, it was reported that female fecundity is reduced by the induction of innate immunity [36] and lesser expressions of AMP genes are found in longer-lived flies [15], suggesting that less activation of AMP synthesis has a beneficial effect for other biological processes. In summary, our data indicate that mild downregulation of the Imd pathway increases stress resistance, lifespan, and fat content in adult flies, which mimics the enhanced stress resistance caused by a lower developmental temperature [3]. There are many reports showing that stress resistance is intensely related to the aging process [20]. Here, we demonstrate that a delicate modulation of the Imd pathway is important for the regulation of stress resistance and lifespan.

## METHODS

### Fly genotypes and aging assay

The *imdP* (P{EPgy2}imd^EY08573^), *AttC*-P (Mi{ET1}AttC^MB05438^), UAS-*DptB*Ri (P{TRiP.HM05186}attP2), *arm*-Gal4, and r4-Gal4 flies were obtained from the Bloomington *Drosophila* stock center. The UAS-dsRNAi stocks (*AttC*Ri: V47041; *imd*Ri: V9253) and *imdNP* (P{GawB}imd^NP1182^) flies were obtained from Vienna and Kyoto stock centers, respectively. The *imd1* (*imd^1^*) was kindly provided by D. Kimbrell [23]. The *y^1^ w^1^* (Bloomington) flies were used as wild-type control and the flies obtained outside were six times isogenized with *y^1^ w^1^* before the stress resistance and aging tests. Virgin flies were collected from a bottle in which larval density was controlled in a standard cornmeal medium, and were used for all fly experiments including stress response, aging, and nutritional content studies [26]. For the aging test, 200 virgin flies (20 flies per vial) were counted and transferred to fresh standard cornmeal vials every 3-4 days [5]. Mean lifespan was calculated from the lifespan curves and averaged with standard error of mean (SEM) from 2 ~ 4 independent experiments.

### Stress response assays

To measure stress responses, 100 newly eclosed flies (20 flies per vial) were kept on a standard cornmeal medium at 25°C for 2 days [3,5,26]. For the starvation test, these 2-day-old adult flies were transferred to new vials (2.5 × 9.3 cm) containing two filter circles (2.4-cm diameter, Fisher Scientific) soaked in 300 μl of distilled water, and were maintained at 25°C under moist conditions with 100 μl of water added every 12 hrs. For the oxidative stress test, the 2-day-old adult flies were starved for 6 hrs at 25°C as described above. The flies were then transferred to new vials containing two filter circles wetted with 300 μl of 20 mM methyl viologen hydrate (Paraquat, Fisher Scientific) in a 5% sucrose solution and maintained at 25°C. For the heat test, the 2-day-old adult flies were transferred to new vials containing standard cornmeal medium and maintained at 37°C with 30% humidity. The median survival times of flies under each stress (starvation, oxidation, or heat) were calculated from the survival curves of 4 ~ 8 independent experiments.

### Nutrients and MnSOD assays

All 20 flies were weighed and homogenized with specific buffers for nutrients (fat, protein, and glycogen: 0.01 M KH_2_PO_4_, 1 mM EDTA pH 7.4) [37] and MnSOD assays (20 mM HEPES, pH 7.2, 1 mM EGTA, 210 mM mannitol, 70 mM sucrose). The supernatant was recovered by centrifugation at 4,000*g* for 10 min at 4°C and used for nutrient measurement (triacylglycerol: Cayman Chemical; protein: Pierce; glycogen: BioVision). For measuring MnSOD activity, the recovered supernatant above was centrifuged again at 10,000*g* for 15 min at 4°C to pellet down the mitochondrial fraction containing MnSOD. This pellet was homogenized with the same buffer and used for the MnSOD assay (Cayman Chemical) in the presence of 2 mM KCN (Cu/ZnSOD inhibitor). Each assay was repeated and averaged (± SEM) from the 5 ~ 20 independent samples.

### Microarray and RT-PCR analyses

Total RNA was extracted with the TRIzol Reagent (Invitrogen) from a whole body of male adult flies and used as probes for the hybridization reaction on Affymetrix *Drosophila* 2.0 microarray chips in the EOHSI facility (http://eohsi.rutgers.edu/). The expression levels of genes were calculated by the RMA (robust multichip analysis) normalization method. For RT-PCR, oligo dT-primed cDNAs (Superscript II RT, Invitrogen) were made from 5 μg total RNA of adult flies and used as templates for the quantitative real-time PCR, which was performed with power SYBR green PCR mix (Applied Biosystems). The *rp49* gene was used as an internal reference for normalizing the quality of total RNA purified from each flies. The fold changes in gene expression were determined by comparative C_T_ method (ABI Prism 7700 Sequence Detection System User Bulletin #2, Applied Biosystems) and then, were averaged with SEM from 4 ~ 8 independent experiments using at least two different RNA batches.

## Supplementary Material

Supplementary File
