# Supplementary material for: Stress resistance and lifespan enhanced by downregulation of antimicrobial peptide genes in the Imd pathway
Source: Aging (Albany NY). 2018 Apr 19;10(4):622–31. doi: 10.18632/aging.101417 (PMC5940113; doi:10.18632/aging.101417)
Supplement: Supplementary File [file aging-10-101417-s001.pdf]

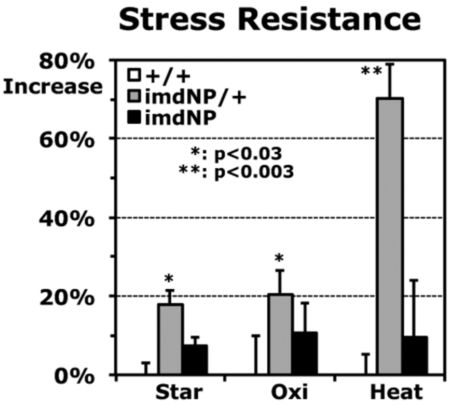

Supplementary Figure S1. Stress resistance enhanced by 2-day-old *imdNP* mutant flies developed at 25°C. The median survival times of wild-type control flies (+/+: 0%) were 34.0 hours in starvation, 18.5 hours under oxidation, and 10.5 hours with heat stress.

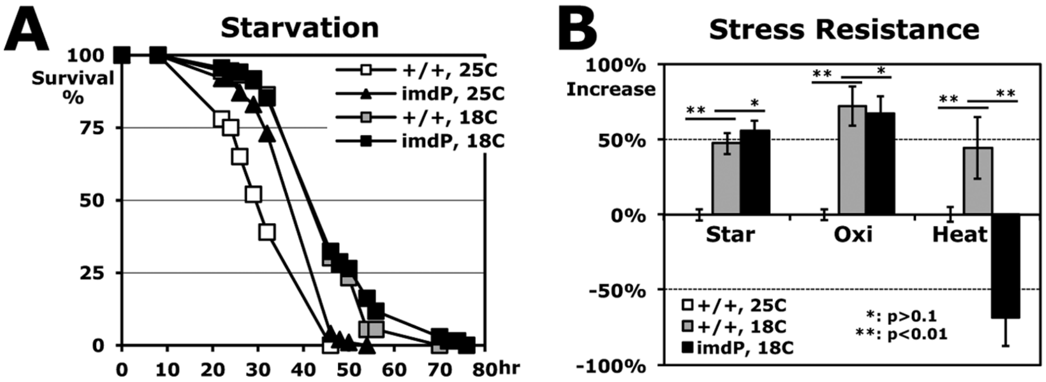

Supplementary Figure S2. Stress resistance of *imdP* mutant flies developed at 18°C. (**A**) The survival curve for starvation stress using 2-day-old male flies developed at 25°C or 18°C. (**B**) Comparison of stress resistance between wild-type and *imdP* mutant flies developed at 18°C. The changed percentage of median survival times under each stress (starvation, oxidation or heat) were calculated from wild-type flies developed at 25°C (+/+, 25C: 0%).
